# Selection of Reliable Reference Genes for Gene Expression Studies Using Real-Time PCR in Tung Tree during Seed Development

**DOI:** 10.1371/journal.pone.0043084

**Published:** 2012-08-17

**Authors:** Xiaojiao Han, Mengzhu Lu, Yicun Chen, Zhiyong Zhan, Qinqin Cui, Yangdong Wang

**Affiliations:** 1 State Key Laboratory of Tree Genetics and Breeding, Chinese Academy of Forestry, Beijing, People's Republic of China; 2 Research Institute of Subtropical Forestry, Chinese Academy of Forestry, Fuyang, People's Republic of China; 3 Research Institute of Forestry, Chinese Academy of Forestry, Beijing, People's Republic of China; Kyushu Institute of Technology, Japan

## Abstract

Quantitative real-time PCR (RT-qPCR) has become an accurate and widely used technique to analyze expression levels of selected genes. It is very necessary to select appropriate reference genes for gene expression normalization. In the present study, we assessed the expression stability of 11 reference genes including eight traditional housekeeping genes and three novel genes in different tissues/organs and developing seeds from four cultivars of tung tree. All 11 reference genes showed a wide range of Ct values in all samples, indicating that they differently expressed. Three softwares – geNorm, NormFinder and BestKeeper – were used to determine the stability of these references except for *ALB* (2S albumin), which presented a little divergence. The results from the three softwares showed that *ACT7* (Actin7a), *UBQ* (Ubiquitin), *GAPDH* (glyceraldehyde-3-phosphate dehydrogenase) and *EF1α* (elongation factor 1-α) were the most stable reference genes across all of the tested tung samples and tung developing seeds, while *ALB* (2S albumin) was unsuitable as internal controls. *ACT7*, *EF1β* (elongation factor1-beta), *GAPDH* and *TEF1* (transcription elongation factor 1) were the top four choices for different tissues/organs whereas *LCR69* did not favor normalization of RT-qPCR in these tissues/organs. Meanwhile, the expression profiles of *FAD2* and *FADX* were realized using stable reference genes. The relative quantification of the *FAD2* and *FADX* genes varied according to the internal controls and the number of internal controls. The results further proved the importance of the choice of reference genes in the tung tree. These stable reference genes will be employed in normalization and quantification of transcript levels in future expression studies of tung genes.

## Introduction

Tung tree (*Vernicia fordii* Hemsl.), a subtropical round-crowned deciduous tree, belongs to a species of the genus *Vernicia* in the spurge (Euphorbiaceae) family. Tung oil extracted from seeds is considered to be one of the high-value industrial oils [1], used widely in production of cloth, shoes, waterproofing masonry, clothing, paper, and biodiesel [1], [2]. With 80% α-eleostearic acid of tung oil, a high degree of unsaturation, tung oil is regarded as a conjugated drying oil [3], [4]. Following the development and maturation of tung tree seeds, the content of fatty acids gradually increases. The peak periods of fatty acid synthesis are during the middle of August and the middle of September in China [5]. The construction of cDNA library of tung seeds and the release of expressed sequence tag (EST) databases have greatly promoted the study of genes involved in fatty acids synthesis such as delta-12 fatty acid desaturase (*FAD2*), delta 12 fatty acid conjugase (*FADX*), diacylglycerol acyltransferase 1 (*DGAT1*) and diacylglycerol acyltransferase 2 (*DGAT2*) [6], [7]. Therefore, the understanding of expression patterns of some key genes will help elucidate the mechanism involved in fatty acids synthesis of tung seeds.

Methods to detect gene expression level include Northern blot, semi-quantitative PCR (semi-PCR), RNase protection analysis (RPA), gene chips and quantitative real-time PCR (RT-qPCR). RT-qPCR has become a very powerful method to detect and quantify gene transcription levels due to its high sensitivity, specificity, reproducibility and accuracy [8]–[10]. Besides, in many situations, it is the only method for detecting mRNA levels of low copy number target genes of interest [11]. However, the factors of RNA stability, quality, quantity, retrotranscription efficiencies and PCR reaction can affect the reliability of testing result for RT-qPCR [8], [12]. Thus, normalization for transcript levels of test genes is crucial to minimize technical variations [12].

One of the methods used to normalize RT-qPCR data is to select appropriate reference genes for controlling the experimental possible errors generated during the detection process [12]. Ideal reference genes are expected to be stable at an expression level across various experimental conditions such as plant developmental stages, tissue types and external stimuli [13]. The most commonly used reference genes include β-actin (*ACT*), glyceraldehyde-3-phosphate dehydrogenase (*GAPDH*), 18S ribosomal RNA (*18S rRNA*), 25S ribosomal RNA (*25S rRNA*), polyubiquitin (*UBQ*), ubiquitin conjugating enzyme (*UBC*), translation elongation factor (*TEF*), cyclophylin (*CYC*), elongation factor 1-α (*EF1α*) and tubulin (*TUB*) etc. [8], [14], [15]. However, recent studies have shown that some of these references might not be stably expressed under different experimental conditions [16]. For example, *UBC16* expression in leaves of the lily plants was quite stable under various treatments, whereas its expression was rather variable in the roots [17]. In Chinese cabbage, *EF1α* is the most suitable reference genes among different tissues, but *GAPDH* is the best choice for experiment under conditions of drought stress and downy mildew infection [18]. Therefore, it is necessary to screen and identify novel reference genes from expressed sequence tags databases (EST), transcriptome data, Microarray analysis and cDNA libraries [19]. *Expressed1*, *SNAD* and *CACS* from transcriptome sequence data in buckwheat, for instance, are revealed as the most stable in different plant structures [20].

As far as is known, selection and identification of stable reference genes refer to many plant species and cultivated varieties [19]. In the tung tree, albumin *(ALB)* and ubiquitin ligase *(UBC)* have been used as reference genes in developing seeds [6], [21]; however, the stability of both genes has not yet been accessed. Thus, identification of reliable reference genes for RT-qPCR will benefit further studies on the tung seeds development and different tissue/organs at the transcription level. In the present study, we aimed to identify potential reference genes suitable for transcript normalization in tung developing seeds and different tissue/organs. The expression profiles of 11 reference genes including *ACT7* (actin7a), *ALB* (2S albumin), *EF1α* (elongation factor1-alpha), *EF1β* (elongation factor1-beta), *TEF1* (transcription elongation factor 1), *GAPDH* (glyceraldehyde-3-phosphate dehydrogenase), *LCR69* (low molecular weight cysteine-rich 69), *SAMDC* (S-adenosylmethionine decarboxylase), *TCTP* (translationally controlled tumor protein), *UBC* (ubiquitin-conjugating enzyme E2) and *UBQ* (ubiquitin), were studied in seven different tissue/organs and six different development stages of seeds collected from four cultivars of tung tree, and the expression stability of these genes was subsequently evaluated using geNorm [22], Bestkeeper [23] and NormFinder [24]. Furthermore, the expression patterns of two target genes *FAD2* and *FADX* were investigated using the selected references, which may be helpful to reveal their roles in fatty acids synthesis.

## Results

### Verification of amplicons, primer specificity, Ct data collection and gene-specific PCR amplification efficiency

**Figure 1:**
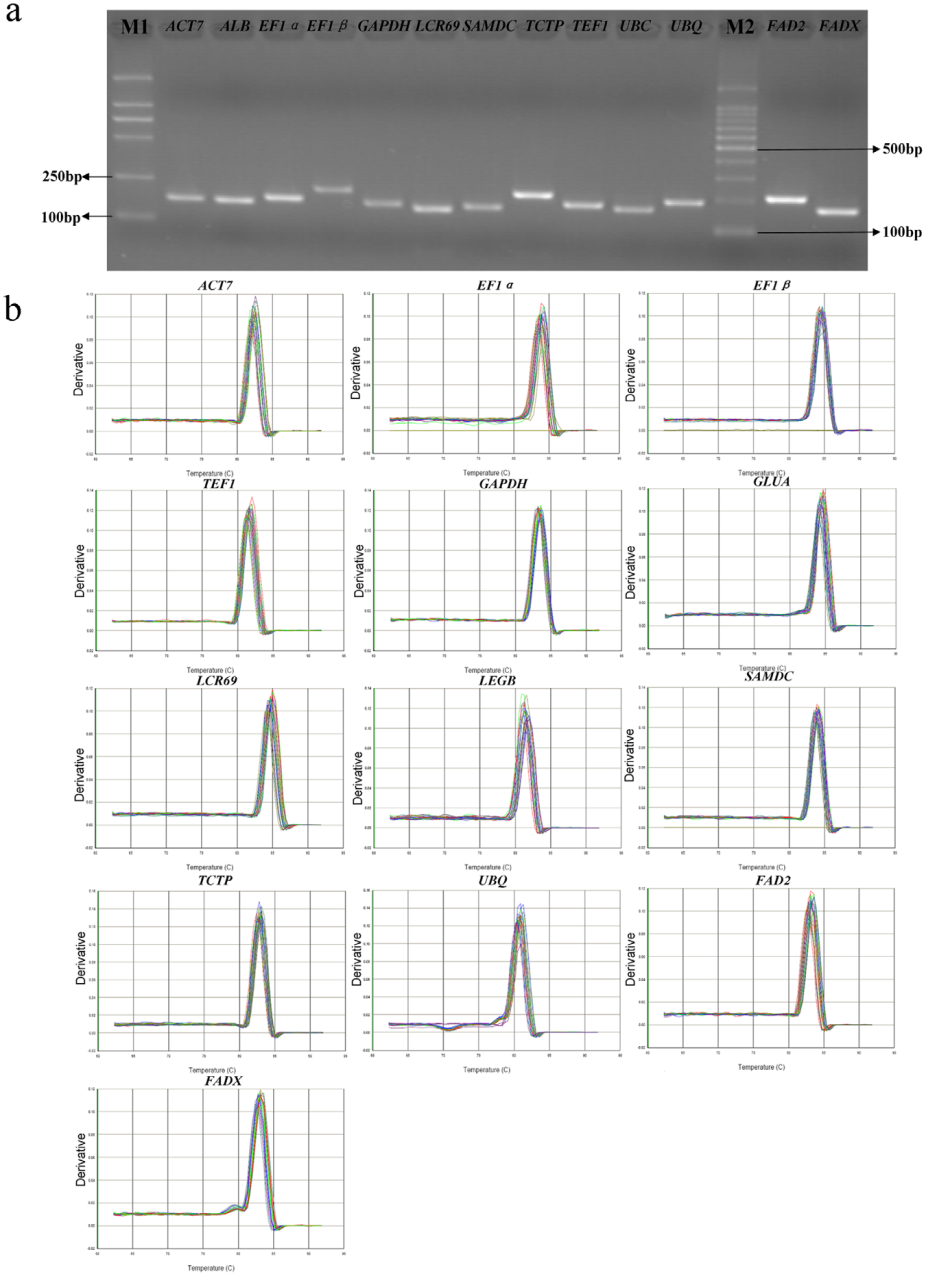
Gene specificity and amplicon size. (a) Agarose gel (2%) electrophoresis showing amplification of a specific PCR product of the expected size for each gene. (b) Melting curves of 11 reference genes and 2 target genes showing single peaks. M1 and M2 represent 2000 bp and 100 bp DNA ladder marker respectively.

A total of 11 reference genes from the tung tree kernel uncut cDNA library were selected as candidates for normalization of gene expression measures. Gene name, accession number, gene description, primer sequences, amplicon length, amplification efficiency, Tm values and correlation coefficients were listed in Table 1. The melting temperatures (*Tm*) of all PCR products ranged from 80.71°C for *UBQ* to 84.85°C for *LCR69*. Amplification efficiency (*E*) of PCR reactions varied from 93.40% for *UBC* to 100.03% for *ACT7*, and correlation coefficients (*R^2^*) ranged between 0.9984 for *ACT7* and 1 for *LCR69*, respectively (Table 1). The amplifications were confirmed by the presence of a single band of expected size for each primer pairs in 2% agarose gel electrophoresis (Figure 1a) and by the single peak melting curves of the PCR products (Figure 1b). No primer dimers or other PCR products were generated from non-specific amplification (Figure 1a), and no products were detected in negative controls.

**Table 1:** Candidate reference genes, primers and different parameters derived from RT-qPCR analysis.

Gene Symbol	Gene name	GenBank accession number	Primer sequence(5′→3′) (forword/reverse)	Tm (°C)	Amplicon Length (bp)	Amplification efficiency (%)	R^2^
*ACT7*	Actin7a	JQ680035	CGATGAAGCACAGTCCAAAAGGTTGAGAGGAGCCTCAGTG	82.36	170	100.03	0.9984
*ALB*	2S albumin	JQ680046	TAAGGCAACAAATGGCTTCCACATCGAAACCCTGAAGACG	87.06	166	97.91	0.9997
*EF1α*	Elongation factor 1-alpha	JQ680036	GCCTGGTATGGTTGTGACCT GGATCATCCTTGGAGTTGGA	84.01	180	97.2	0.9995
*EF1β*	Elongation factor 1-beta	JQ680037	CGAATCAGGCCTCAAGTCTC CACCTTTGCCACCAATTCTT	84.72	218	98.14	0.9992
*GAPDH*	Glyceraldehyde-3- phosphate dehydrogenase	JQ680038	CTGCTAAGGCTGTTGGGAAG TCCCTCTGACTCCTCCTTGA	83.55	168	97.41	0.9999
*LCR69*	Low molecular weight cysteine-rich 69	JQ680039	CCTCCTCTTCTTGCTGCTTG GTAACCCTCGGCAGTCTCCT	84.85	155	96.15	1
*SAMDC*	S-adenosylmethionine decarboxylase	JQ680043	CCTGGAGCTCAGTCGTATCC CCAAACCAGTCATGCACATC	83.16	208	93.53	0.9987
*TCTP*	Translationally controlled tumor protein	JQ680040	GAAGGGGCAGATGAAGATGAGAGAGCAGGAACTTGGTTGC	82.95	214	95.58	0.9996
*TEF1*	Transcription elongation factor 1	JQ680042	GTTGTCCCTTCTGCAACCAT AACGTTGTTAACCCGCTCAC	81.48	179	95.01	0.9997
*UBC*	Ubiquitin-conjugating enzyme E2	JQ820248	CCATTTCCAAGGTGTTGCTT GGCAGCACTGTTAACCCATT	83.31	165	93.40	0.9993
*UBQ*	Ubiquitin	JQ680041	CCGTGGTGGCTGTTAAGTTT AAGGCCATTTCAACATCCTG	80.71	193	94.47	0.9993
*FAD2*	delta-12 fatty acid desaturase	AF525534	AGCATCCGCTGGTTCTCTAA GCAAGAACACCAGCATCAGA	83.25	212	99.87	0.9994
*FADX*	delta 12 fatty acid conjugase	AF525535	GGAAAGCAGAAGCGTGAAACAGGTGGTGGCAATGGAGTAG	83.01	170	94.7	0.9991

**Figure 2:**
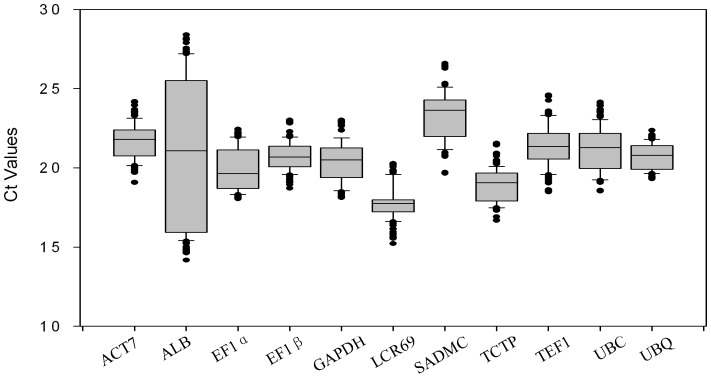
RT-qPCR Ct values for reference genes. Expression data displayed as Ct values for each reference gene in all tung samples. A line across the box is depicted as the median. The box indicates the 25th and 75th percentiles, whisker caps represent the maximum and minimum values, dots represent outliers.

The cycle threshold (Ct) values were obtained from each reaction with 11 primer pairs. To reveal the differences in transcript levels between 11 reference genes, it is necessary to assess the Ct range and calculate the coefficient of variance for each gene across all samples. As expected, the average Ct values varied between the different genes ranging from 16 to 27 cycles (Figure 2). *LCR69* with narrow variance was the most abundant reference transcript, reaching threshold fluorescence only 17 to 19 amplification cycles, while *SADMC* was the least abundant transcript with Ct of 24. The expression stability was also detected by calculating coefficient of variance (CV) of Ct values. A seed storage protein *ALB* had large variances in their transcript levels, and the CV values were more than 11 for all samples, indicating that the gene was unstable. On the other hand, *EF1β*, *GAPDH*, *TEF* and *UBQ* had narrow variances in their transcript expressions.

### Expression stability of candidate reference genes

Three different software programmes were used to evaluate the expression stability of the candidate reference genes: geNorm [22], Bestkeeper [23] and NormFinder [24]. Ct data were collected across all samples. Ct values were used directly for stability calculations for BestKeeper analysis, and these data were transformed to relative quantities using the delta-Ct method for geNorm and NormFinder analysis.

#### a) geNorm analysis

**Figure 3:**
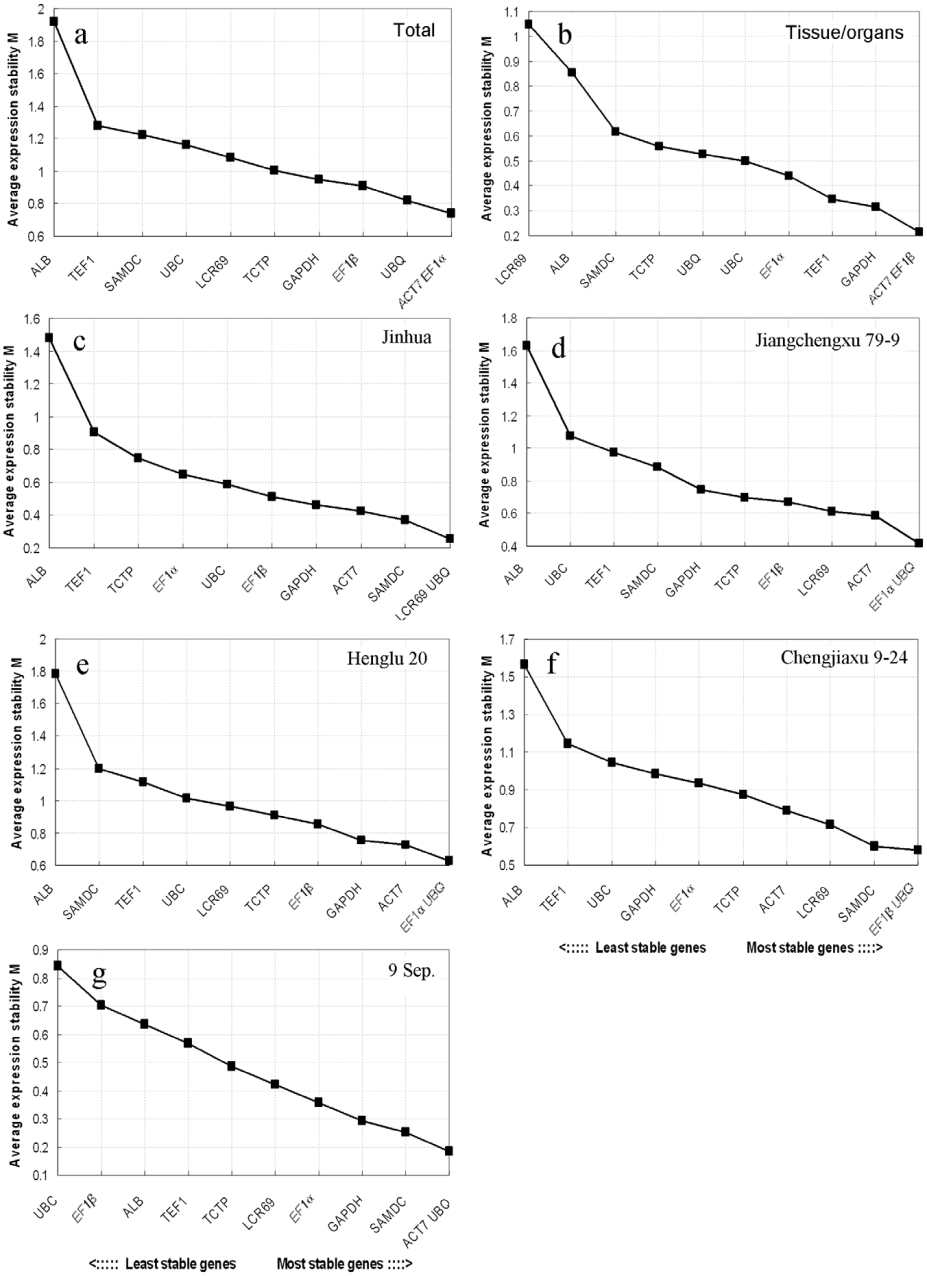
Average expression stability values (M) calculated by geNorm. (a) all 31 samples, (b) different tissue/organs, (c) the cultivar “Jinhua”, (d) the cultivar “Jiangchengxu 79-9”, (e) the cultivar “Henglu 20”, (f) the cultivar “Chengjiaxu 9–24”, (g) four cultivars from 9 September. Lower average expression stability (M value) indicates more stable expression.

geNorm was used to rank the reference genes by calculating gene expression stability value *M* based on the average pairwise expression ratio. The most stable reference gene has the lowest *M* value, while the least stable one presents the highest *M* value. The program recommends using *M* value below the threshold of 1.5 to identify reference genes with stable expression. In our analysis of four cultivars, all genes except *ALB* had *M* <1.5 as a criterion to consider the tested genes as rather stable (Figure 3). When all the results from all 31 samples of *V. fordii* were combined, *EF1α* and *ACT7* had the highest expression stability (the lowest *M* value), whereas a seed storage protein *ALB* was revealed less stability and other eight genes were placed at the intermediate positions between the two extremums (Figure 3a). Stability rank of the 11 tested reference genes in the seven tissue/organs confirmed that all genes had *M* values below the threshold of 1.5, and *EF1β* and *ACT7* had the highest expression stability (Figure 3b). For the two cultivars “Jiangchengxu 79-9” and “Henglu 20”, *EF1α* and *UBQ* were the most stably expressed genes (Figure 3d and Figure 3e). In contrast, for the cultivar “Jinhua”, *LCR69* and *UBQ* were the most stably expressed genes (Figure 3c). Besides, the geNorm analysis also indicated that *EF1β* and *UBQ* were the most stably expressed reference genes in the cultivar “Chengjiaxu 9–24” (Figure 3f). Therefore, there was a little change of the reference ranks in four different cultivars. Furthermore, reference gene expression stability was analyzed in the samples of four different cultivars from 9 September (Figure 3g). *ACT7*and *UBQ* had the lowest *M* value with the highest expression stability. Overall, all of the tested reference genes except *ALB* showed relatively high stability with low *M* values of less than 1.3 (Figure 3).

**Figure 4:**
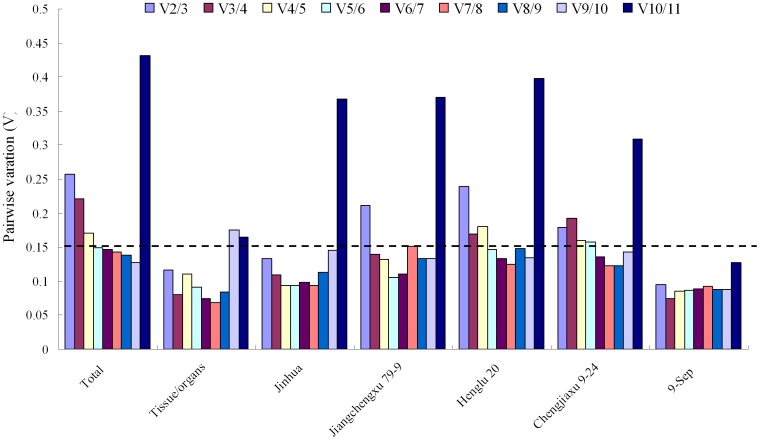
Pairwise variation (V) calculated by geNorm to determine the optimal number of reference genes. The average pairwise variations Vn/Vn+1 was analyzed between the normalization factors NFn and NFn+1 to indicate the optimal number of reference genes required for RT-qPCR data normalization in different samples.

To evaluate the optimal number of reference genes required for accurate normalization, the pairwise variation (Vn/Vn+1) was calculated using geNorm between consecutively ranked normalization factors. Generally, 0.15 was used as a cutoff value to determine the optimal number of reference genes [22]. In our data sets, the paired variable coefficient in all samples indicated that the inclusion of the fourth reference gene hardly contributed to the variation of the normalization factor, whereas two stable reference genes *ACT7* and *EF1β* or *ACT7*and *UBQ* (V2/3<0.15) in different tissue/organs or different cultivars from 9 September would be sufficient for normalizing gene expression (Figure 4). When all 31 samples were taken together to determine the number of reference genes, the pairwise variation of V2/3 was higher than 0.15 (0.257), as were V3/4 (0.221) and V4/5 (0.171). The V5/6 value was 0.148, indicating that at least five reference genes should be included for gene expression studies in all the samples of *V. fordii*.

#### b) NormFinder analysis

NormFinder is another Excel application, which ranks the candidate genes according to stability index M based on the average pairwise variation of a gene compared to the rest of the studied genes [22]. The more stably expressed genes exhibited the lower average expression stability values (M values). The stability value of each gene was calculated by NormFinder as shown in Table 2. This analysis method identified that *ACT7*, *EF1β*, *UBQ* and *GAPDH* were the most appropriate for use as a reference gene in all samples. For the different tissues/organs and the cultivar “Jiangchengxu 79-9”, *GAPDH*, *TEF1*, *UBQ* and *ACT7* had the most stable expression. In the three cultivars “Jinhua”, “Henglu 20”, “Chengjiaxu 9–24”, *LCR69*, *UBQ*, *GAPDH* and *ACT7* had the most stable expression and were the ideal reference genes. In four cultivars collected from 9 September, *ACT7* and *UBQ* were the stable reference genes. For all tested samples, *ALB* was the least stable reference genes. Due to unstable expression according to the results of geNorm and NormFinder analysis, the candidate *ALB* was discarded from subsequent analysis.

**Table 2:** Ranking of candidate reference genes in order of their expression stability as calculated by NormFinder.

Rank	Total	Tissue/organs	Jinhua	Jiangchengxu 79-9	Henglu 20	Chengjiaxu 9–24	9 Septmber
1	*ACT7* (0.121)	*GAPDH* (0.023)	*LCR69* (0.089)	*ACT7* (0.054)	*UBQ* (0.066)	*UBQ* (0.178)	*ACT7* (0.064)
2	*EF1β* (0.341)	*TEF1* (0.265)	*UBQ* (0.191)	*GAPDH* (0.321)	*GAPDH* (0.192)	*EF1β* (0.202)	*UBQ* (0.064)
3	*UBQ* (0.403)	*UBQ* (0.277)	*ACT7* (0.206)	*UBQ* (0.341)	*ACT7* (0.299)	*LCR69* (0.340)	*SAMDC* (0.175)
4	*GAPDH* (0.438)	*ACT7* (0.292)	*SAMDC* (0.222)	*TEF1* (0.456)	*TEF1* (0.491)	*ACT* (0.447)	*GAPDH* (0.212)
5	*TEF1* (0.459)	*UBC* (0.297)	*GAPDH* (0.229)	*SAMDC* (0.464)	*EF1β* (0.493)	*SAMDC* (0.474)	*EF1α* (0.326)
6	*EF1α* (0.593)	*TCTP* (0.299)	*EF1β* (0.328)	*EF1α* (0.504)	*EF1α* (0.520)	*GAPDH* (0.519)	*LCR69* (0.362)
7	*LCR69* (0.697)	*EF1α* (0.335)	*UBC* (0.552)	*LCR69* (0.552)	*TCTP* (0.776)	*TEF1*(0.711)	*TEF1* (0.419)
8	*TCTP* (0.712)	*EF1β* (0.347)	*EF1α* (0.641)	*EF1β* (0.617)	*LCR69* (0.813)	*TCT* (0.789)	*TCTP* (0.460)
9	*SAMDC* (0.909)	*SAMDC* (0.627)	*TEF1* (0.670)	*TCTP* (0.847)	*SAMDC* (0.862)	*EF1α* (0.841)	*ALB* (0.530)
10	*UBC* (0.979)	*ALB* (1.155)	*TCTP* (0.975)	*UBC* (0.923)	*UBC* (0.962)	*UBC* (0.935)	*EF1β* (0.651)
11	*ALB* (3.336)	*LCR69* (1.248)	*ALB* (2.804)	*ALB* (2.802)	*ALB* (3.030)	*ALB* (2.351)	*UBC* (0.972)

#### c) Bestkeeper analysis

Bestkeeper, an Excel-based software tool, evaluates the most stably expressed genes based on the coefficient of correlation (r) to the BestKeeper index by calculating the Ct set standard deviation (SD) and coefficient of variance (CV). BestKeeper analysis revealed that the best correlations were obtained for *ACT7* (0.960), *EF1α* (0.883), *GAPDH* (0.892) and *UBQ* (0.829) with *P* value of 0.001 for all samples (Table 3). In the four cultivars, *ACT7*, *UBQ*, *EF1α* and *GAPDH* showed the highest correlations. The results of BestKeeper analysis showed little differences from those obtained from geNorm and Normfinder.

**Table 3:** Statistics results by BestKeeper software for ten selected genes based on Ct values.

coeff. of corr. [r] (*p*-value)	*ACT7*	*EF1α*	*EF1β*	*GAPDH*	*LCR69*	*SAMDC*	*TCTP*	*TEF1*	*UBC*	*UBQ*
Total	0.960 (0.001)	0.883 (0.001)	0.774 (0.001)	0.892 (0.001)	0.435 (0.015)	0.725 (0.001)	0.626 (0.001)	0.577 (0.001)	0.641 (0.001)	0.829 (0.001)
Tissue/organs	0.980 (0.001)	0.861 (0.013)	0.980 (0.001)	0.980 (0.001)	0.153 (0.741)	0.840 (0.018)	0.896 (0.006)	0.985 (0.001)	0.884 (0.008)	0.847 (0.016)
Jinhua	0.903 (0.014)	0.786 (0.064)	0.743 (0.091)	0.816 (0.048)	0.842 (0.036)	0.897 (0.015)	0.761 (0.079)	0.283 (0.587)	0.761 (0.079)	0.093 (0.003)
Jiangchengxu 79-9	0.955 (0.003)	0.964 (0.002)	0.718 (0.108)	0.871 (0.024)	0.848 (0.033)	0.674 (0.143)	0.560 (0.248)	0.643 (0.168)	0.459 (0.361)	0.904 (0.013)
Henglu 20	0.988 (0.001)	0.858 (0.029)	0.849 (0.033)	0.928 (0.008)	0.538 (0.27)	0.647 (0.164)	0.711 (0.113)	0.823 (0.044)	0.63 (0.181)	0.917 (0.010)
Chengjiaxu 9–24	0.982 (0.001)	0.942 (0.005)	0.975 (0.001)	0.917 (0.010)	0.947 (0.004)	0.872 (0.024)	0.807 (0.052)	0.657 (0.157)	0.614 (0.194)	0.849 (0.033)

### Reference gene validation

**Figure 5:**
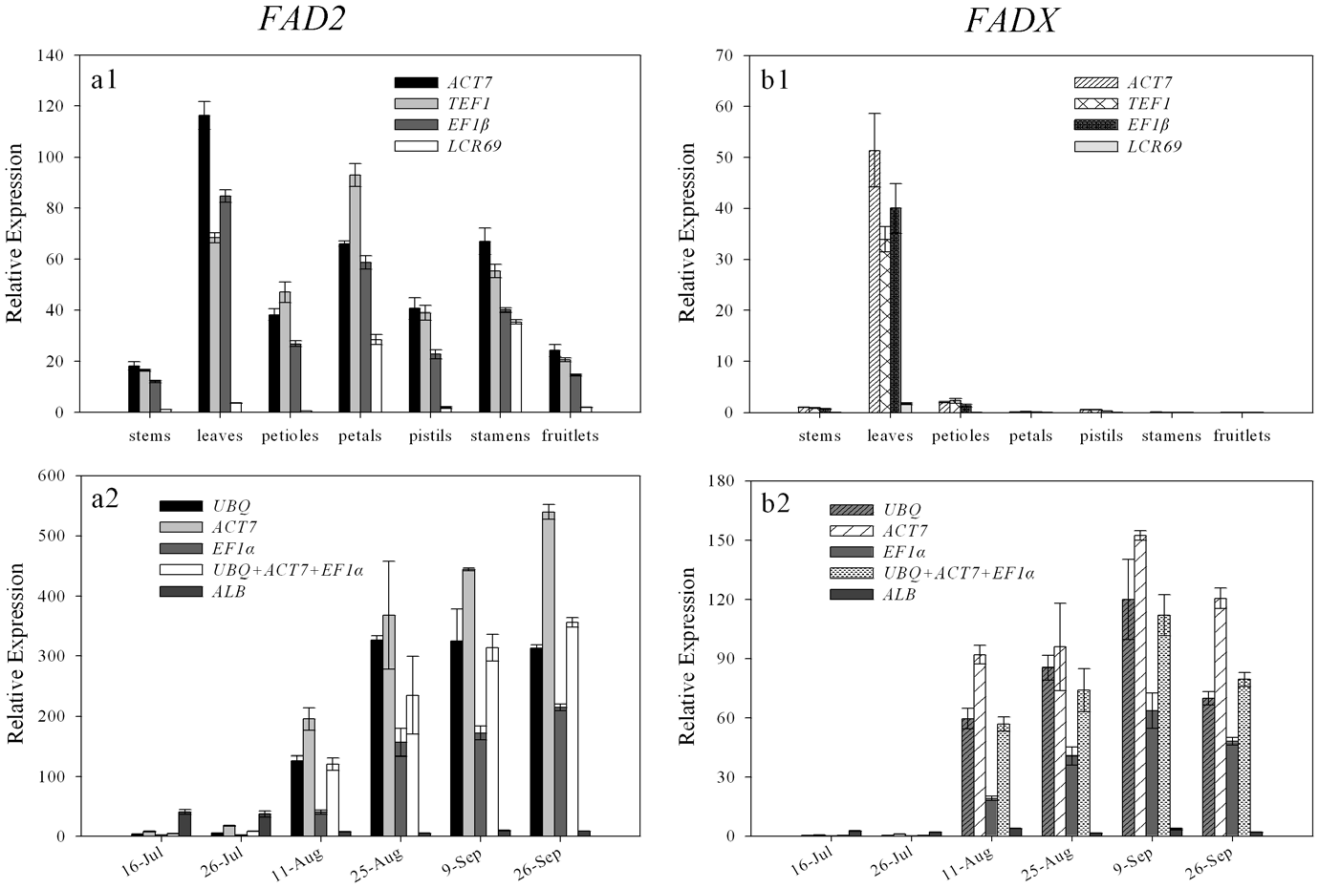
Expression levels of *FAD2* and *FADX* in different tissues/organs and seeds development of the cultivar “Jiangchengxu 79-9”. (a1 and a2) Expression levels of *FAD2* in different tissues/organs and seeds development, (b1 and b2) Expression levels of *FADX* in different tissues/organs and seeds development. Genes were normalized to individual and/or combined reference genes. Error bars show mean standard error calculated from two biological replicates.

The use of different reference genes to calculate relative expression data could have a significant influence on the final normalized results. To detect the effect of reference gene on the outcome of a practical experiment, the relative expression patterns for two functional genes *FAD2* and *FADX* were evaluated using different reference genes in seven different tissue/organs and six different developmental stages of tung seeds from the cultivar “Jiangchengxu 79-9” (Figure 5). The most stable references *ACT7*, *TEF1* and *EF1β* in the different tissue/organs selected by geNorm and Bestkeeper were used as internal controls. *FAD2* was expressed in all tested tissues/organs, with a higher level in leaves and petals (Figure 5a1), while *FADX* expression was restricted to leaves (Figure 5b1). However, *FAD2* and *FADX* were expressed at a lower level when using the least stable reference *LCR69* as internal control. When stable references *UBQ*, *ACT7*, *EF1α*, and the combination of the three references were used as internal controls respectively, the expression patterns of *FAD2* and *FADX* showed a low expression in earlier stages of tung seeds development (16 July and 26 July). Both *FAD2* and *FADX* exhibited a similar expression pattern with an increase from 11 August to 9 September (Figure 5a2 and Figure 5b2). When the least stable reference gene *ALB* was used for normalization, the two target genes were expressed in lower levels in tung developing seeds, and showed a reverse result compared to the stable references for normalization. Thus, the use of unsuitable references can lead to over- or underestimation of relative transcript abundance. These results reinforce the importance of validating reference genes prior to experimental applications.

## Discussion

At present, quantitative real-time PCR has significantly improved the detection and quantification of expression profiles of target genes due to its high throughput, sensitivity, specificity, accuracy and broad quantification range [9], [10]. It is very necessary to screen appropriate internal reference genes for gene expression normalization during target genes expression analyzed. A stable expressed reference gene should produce constant Ct values under different experimental conditions such as plant developmental stages, tissue types and external stimuli [13]. Here, the stability of expression of three novel and eight traditional reference genes was evaluated in different tissue/organs and developing seeds from four different cultivars of tung tree. *EF1β*, *GAPDH*, *TEF*, *UBQ* and *LCR69* with narrow Ct values were stable in the developing seeds. A seed storage protein *ALB* had large variances in their transcript levels, indicating that the reference gene was unstable.

Among recent studies on qRT-PCR, commonly used traditional reference genes, e.g. *ACT*, *GAPDH*, *18S rRNA*, *25S rRNA*, *UBQ*, *UBC*, *TEF*, *CYC*, *EF1α*, *TUB* were considered to be stable and suitable in various tissues [14], [25], since these genes are present in all cell types and necessary for basic cell survival. Nevertheless, numerous researches have already shown that the expression of these traditional genes might also be variational [26]–[28]. Thus, normalization with multiple reference genes is becoming popular and standard in plant research [23], [24]. The present study demonstrates the importance of screening reference genes. geNorm analysis is used to determine the optimal number of stable reference genes for accurate normalization [29]. Generally, 0.15 was used as a cutoff value to confirm the optimal number of reference genes [22]. However, this is not an absolute rule and depends on the dataset tested. A higher V value is considered in other reports [30]–[32]. In the present study, when all samples were taken together to determine the number of reference genes, the pairwise variation of V2/3, V3/4 and V4/5 were higher than 0.15 (Figure 3). The V5/6 value was 0.148, thus the result shows that five genes are included to support gene expression studies. This indicates that the combination of multiple references is necessary to normalize gene expression for all the samples of *V. fordii*.

When gene expression stability in all samples was analyzed by geNorm, the most stable genes were *ACT7* and *EF1α*, followed by *UBQ* (Figure 3). The genes encoding actin, elongation factor 1-alpha and ubiquitin are often considered as reliable reference genes under various experimental conditions. For example, *ACT11* and *EF1α* exhibited a stable expression pattern across different tissues in the water lily [17]. *ACT7* is one of stable references genes for developing embryos in *Brassica napus* [33]. Besides, *ACT* also had the highest expression stability across leaf and root tissues in chicory [34] and tomato [35]. In addition, *EF1α* is a stable reference gene in darnel ryegrass [36], grape berry development [25], different developmental stages and under mild Cd stress conditions in poplar leaves [37], and also in cucumber [38]. *UBQ* exhibited the most stable expression across all samples of *Arabidopsis* [26]. However, *UBQ10* was the most variable reference gene, and should be avoided as an internal control in rice, soybean and the development of grape berry [25], [29], [39]. An ubiquitin tag is reported to mark particular proteins for proteolytic elimination, but it can also have nonproteolytic functions [40], thus its wide range of function lead to the variable expression of ubiquitin in different plants. According to the results of geNorm and NormFinder, a seed storage protein *ALB* was ranked in the last position in all samples and developing seeds from the four cultivars. In different tissue/organs, *ALB* was the least abundant transcript with Ct values of 26–28, indicating that the expression level of the reference gene was very low. When using a stable reference *UBQ* as internal control, *ALB* was not detected in the developing seeds in July. The transcript level rapidly increased from 11 August, and slightly declined on 9 September (data not shown). The results showed that *ALB* was not suitable for reference gene in tung tree. In animals, albumin is present in all nucleated cell types and is necessary for basic cell survival and considered to be stable in various tissues [23]. This indicates that there is a great difference in the expression of *ALB* between plants and animals, thus the reference gene is suitable for animals but not for plants.

The results from geNorm and NormFinder analysis showed some differences, especially in the top ranked genes. However, the output of both programs very consistently listed the same genes showing unstable expression patterns. This little divergence probably reflects differences in the statistical algorithms. It was also reported that there were discrepancies between NormFinder and geNorm in other studies. For example, in *citrus*, the *FBOX/SAND* pair was selected as the least variable among all reference genes by geNorm, but the most stable reference gene according to NormFinder was *UPL7* [41].

To validate the suitability of the reference genes we identified in this study, the expression profiles of *FAD2* and *FADX* were assessed in different tissue/organs and tung developing seeds of the cultivar “Jiangchengxu 79-9”. In tung oil biosynthesis, FAD2 desaturates oleic acid (18∶1Δ^9^) to produce linoleic acid (18∶ 2Δ^9, 12^), then FADX converts linoleic acid to eleostearic acid (18∶3Δ^9, 11, 13^) [42], [43]. The data showed that the use of the most stable reference genes *UBQ, ACT7*, *EF1α* or the combination of stable references resulted in the trend consistency of the relative transcript abundance of *FAD2* and *FADX*. However, the relative transcript abundance presented a reduction when the most variable reference gene *LCR69* or *ALB* used as an internal control (Figure 5). These results suggest that the incorrect use of reference genes may introduce bias in the analysis and lead to the misinterpretation of data.

## Conclusions

In summary, 11 reference genes were evaluated in different tissue/organs and different development stages of tung seeds. We also concluded traditional housekeeping genes that outperformed novel reference genes. The results showed *ACT7*, *UBQ*, *GAPDH* and *EF1α* were suggested as good candidate genes used as reference genes for normalization in gene expression studies. In this constitution, we identify and validate optimal reference genes for RT-qPCR normalization with consideration of different tissues/organs and seed development stages.

## Methods

### Plant materials

Tung fruits of four different cultivars, including “Jinhua,” “Jiangchengxu 79-9,” “Henglu 20” and “Chengjiaxu 9–24” were collected from the National Gene Pool (constructed in 1979) of Tung Tree in Dongfanghong Forest Farm, Zhejiang Province, China. Seven different tissue/organs, including stems, leaves, petioles, petals, pistils, stamens and fruitlets (30 days after flowering) were collected from the cultivar “Jiangchengxu 79-9”. No specific permits were required for the farm to select samples. The farm is not privately-owned in any way and the field studies did not involve protected species. Samples of the six different developmental stages of tung fruits during the increasing periods of fatty acids were taken in 2011: 16 July, 26 July, 11 August, 25 August, 9 September and 26 September. Seeds removed from fruits were immediately frozen in liquid nitrogen and stored at −80°C until needed for RNA extraction. All samples were collected in two replicates.

### RNA extraction and first strand cDNA synthesis

Frozen seeds were hand-shelled, and kernels were ground to a fine powder in liquid nitrogen with a pestle and mortar. About 100 mg of this powder was used for RNA extraction. Total RNA was isolated using the RN38 EASYspin plus Plant RNA kit (Aidlab Biotech, Beijing, China). Purified RNA was quantified with NanoDrop2000 spectrophotometer (Thermo, Wilmington, USA), and loaded on a denaturing 1.0% (p/v) agarose gel to check concentration and integrity. Only RNA samples with 260/280 wavelength ratio between 1.8 and 2.1 and 260/230 wavelength ratio greater than 2.0 were used for cDNA synthesis. cDNA synthesis was performed with 3 µg total RNA using the superscript III first strand synthesis system followed by the RNase H step (Invitrogen, Carlsbad, USA), according to the protocol of the manufacturer in a total volume of 20 µl. cDNAs were diluted 1∶30 with nuclease-free water for RT-qPCR.

### Primer design and PCR conditions

The 11 candidate genes including eight traditional housekeeping genes (*ACT7*, *EF1α*, *EF1β*, *TEF1*, *GAPDH*, *SAMDC*, *UBC* and *UBQ*) and three novel reference genes (*ALB*, *LCR69* and *TCTP*) were selected from the tung tree kernel uncut cDNA library (Table 1). Gene sequences were deposited in the GenBank (accession numbers are listed in Table 1). All reference genes were named based on similarity to known nucleotide sequences using BLAST with a score value higher than 100 and identity ranging from 81% to 94%. Primer pairs were designed using Primer3 (http://frodo.wi.mit.edu/primer3/) with the following parameters: Tm around 60°C and product size range 155–218 base pairs, primer sequences with a length of 19 to 21 nucleotides with an optimum at 20 nucleotides, and a GC content of 45% to 55%. To check all primers specificity, real-time PCR was performed on cDNA and products were analyzed by electrophoresis on 2% agarose gel and ethidium bromide staining.

Real-time PCR reactions were performed in 96-well plates with a 7500 Real Time PCR System (Applied Biosystems, CA, USA) and a SYBR® Premix Ex Taq™ Kit (TaKaRa, Tokyo, Japan) as described by Phillips et al. [44]. PCR reactions were prepared in 20 µl volumes containing 2 µl of 30-fold diluted synthesized cDNA, 10 µl 2× SYBR® Premix Ex Taq™, 0.4 µl 10 µM forward primer, 0.4 µl 10 µM reverse primer, 0.4 µl 50× RO× reference dye and 6.8 µl sterile distilled water. Negative PCR control with no templates was performed for each primer pair. The cycling conditions were recommended by the manufacturer (30 s at 95°C, 40 cycles of 95°C for 5 s, and 60°C for 34 s). Specificity of amplicons was verified by melting curve analysis (60 to 95°C) after 40 PCR cycles. The final threshold cycle (Ct) values were the mean of eight values including two biological replicates for each treatment and four technical replicates.

### Analysis of gene expression stability

Standard curves were constructed to calculate the gene-specific PCR efficiency from 10-fold series dilution of the mixed cDNA template for each primer pair. The correlation coefficients (*R^2^*) and slope values can be obtained from the standard curve, and the corresponding PCR amplification efficiencies (*E*) were calculated according to the equation *E* = (10^−1/slope^−1)×100 [45].

Gene expression stability was evaluated by applying three different statistical approaches: geNorm (ver. 3.5) [22], Bestkeeper (ver. 1.0) [23] and NormFinder (ver. 0.953) [24]. Real-time RT-qPCR data was exported into an Excel datasheet (Microsoft Excel 2003) and Ct values were converted according to the requirements of the software. Each of these approaches generates a measure of reference gene stability, which can be used to rank the order of stability for reference genes.
